# Supplementary material for: Temporal coordination of the transcription factor response to H2O2 stress
Source: Nat Commun. 2024 Apr 23;15:3440. doi: 10.1038/s41467-024-47837-w (PMC11039679; doi:10.1038/s41467-024-47837-w)
Supplement: Supplementary file 1 — Supplementary Information [file 41467_2024_47837_MOESM1_ESM.pdf]

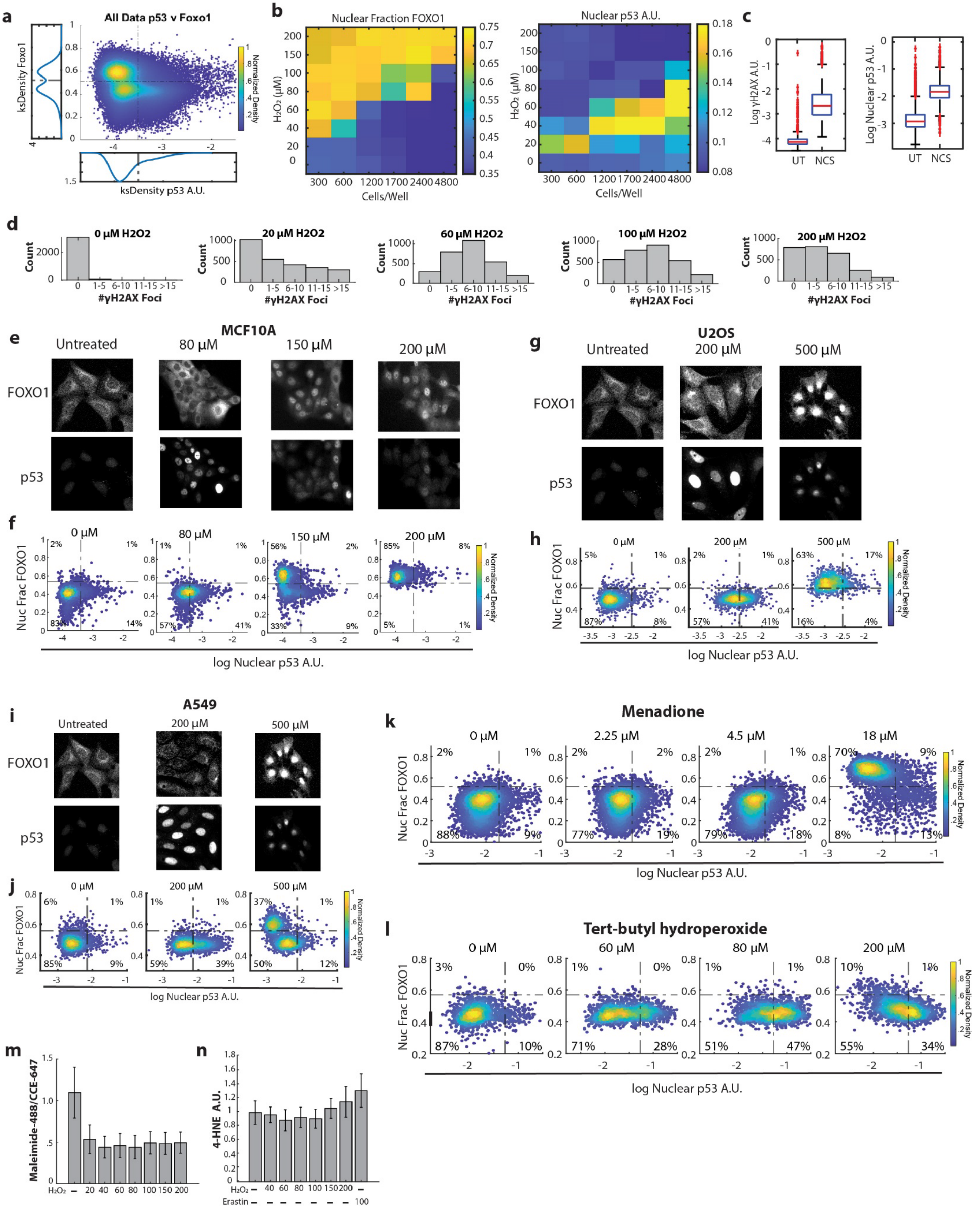
a
All Data p53 v Foxo1
ksDensity Foxo1
ksDensity p53 A.U.
Normalized Density
b
Nuclear Fraction FOXO1
Nuclear p53 A.U.
H₂O₂ (μM)
Cells/Well
c
Log γH2AX A.U.
Log Nuclear p53 A.U.
UT
NCS
d
0 μM H2O2
20 μM H2O2
60 μM H2O2
100 μM H2O2
200 μM H2O2
Count
#γH2AX Foci
e
MCF10A
Untreated
80 μM
150 μM
200 μM
FOXO1
p53
f
Nuc Frac FOXO1
log Nuclear p53 A.U.
g
U2OS
Untreated
200 μM
500 μM
h
i
A549
j
k
Menadione
0 μM
2.25 μM
4.5 μM
18 μM
l
Tert-butyl hydroperoxide
60 μM
80 μM
m
Maleimide-488/CCE-647
H₂O₂
n
4-HNE A.U.
Erastin

**Supplemental Figure 1: Mutually exclusive activation of FOXO1 and p53 in other cell lines. a** Density colored scatter plot of all cells used in Figure 1A-C. Dashed lines indicate thresholds for p53 and FOXO1 activation. Population density plots of nuclear fraction of FOXO1 (left) and p53 (below) used for thresholding data are shown. **b** Heat maps showing the mean nuclear fraction of FOXO1 (left) and mean p53 levels (right) of cells plated at specified cell numbers per well of a 96-well plate (x-axis) and indicated concentrations of $H_2O_2$ (y-axis). **c** Bar and whisker plots of nuclear γH2AX (left) and nuclear p53 levels (right) after 3 hours of treatment with 400ng/mL of Neocarzinostatin (NCS), n>3000 cells. Central line is median, bottom and top edges of box are 25$^{th}$ and 75$^{th}$ percentiles. Whiskers indicate extreme data points. Red pluses are outliers. **d** Histograms of γH2AX foci per cell, n>2000 cells. Measured by immunofluorescence. **e** Immunofluorescence images of MCF10A cells treated with $H_2O_2$ and stained for FOXO1 (top) and p53 (bottom). **f** Density colored scatter plots of log p53 (x-axis) and the nuclear fraction of FOXO1 (y-axis) in MCF10A cells (n>1500 cells) treated with $H_2O_2$. **g** Immunofluorescence images of U2OS cells treated with $H_2O_2$ and stained for FOXO1 (top) and p53 (bottom). **h** Density colored scatter plots of log p53 levels (x-axis) and the nuclear fraction of FOXO1 (y-axis) in U2OS cells (n>900) treated with $H_2O_2$. **i** Immunofluorescence images of A549 cells treated with $H_2O_2$ and stained for FOXO1 (top) and p53 (bottom). **j** Density colored scatter plots of log p53 levels (x-axis) and the nuclear fraction of FOXO1 (y-axis) in A549 cells (n>2000) treated with $H_2O_2$. **k** Density colored scatter plots of log p53 levels (x-axis) and the nuclear fraction of FOXO1 (y-axis) in MCF7 cells (n>5000) treated with Menadione for 5 hours. Data measured by immunofluorescence. **l** Density colored scatter plot of log p53 levels (x-axis) and the nuclear fraction of FOXO1 (y-axis) in MCF7 cells (n>1000) treated with tert-butyl hydroperoxide concentrations. Data measured by immunofluorescence. **m** Median ratio of maleimide-488, and CCE-647. Measured by immunofluorescence. $H_2O_2$ treatment was for 3 hours. Error bars are the median absolute deviation, n>2000 cells. **n** Median 4-HNE staining. Cells were treated with $H_2O_2$ for 3 hours. 100 μm erastin treatment for 24 hours, n>500 cells. All immunofluorescence experiments were repeated three times with similar results. Source data are provided in Source Data Supp Figure 1.

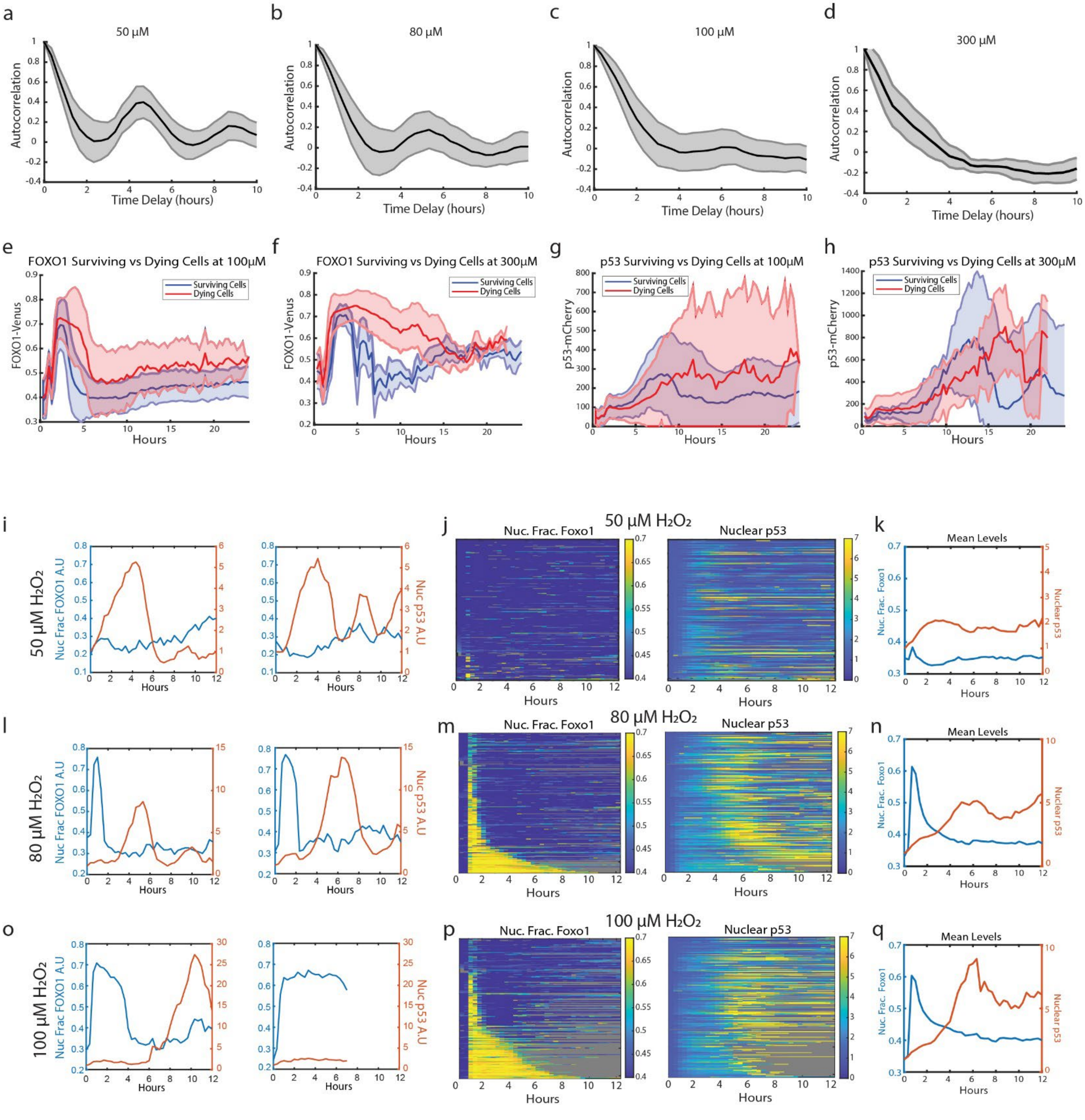


**Supplemental Fig 2: FOXO1 and p53 dynamics in response to $H_2O_2$ stress**. **a-d** Median autocorrelation of p53 trajectories of cells treated with **a** 50 µM (n=188), **b** 80 µM (n=238), **c** 100 µM (n=288) and **d** 300 µM (n=186) $H_2O_2$. **e-f** FOXO1-mVenus levels in surviving (blue) and dead (red) cells treated with **e** 100µM (n=190 living, 98 dead), and **f** 300 µM (n=6 living, 180 dying) of $H_2O_2$ over 24 hours. **g-h** p53-mCherry levels in surviving (blue) and dead (red) cells treated with **g** 100 µM, and **h** 300 µM of $H_2O_2$ over 24 hours. Data in a-h is from MCF7 cells and is the same dataset used in Figure 2. Shaded area in a-h plots represent the median absolute deviation. **i,l,o** Two representative single-cell traces of nuclear fraction of FOXO1-mVenus (blue, left y-axis) and nuclear p53-mCherry om MCF10A cells treated with **i** 50 µM, **l** 80 µM and **o** 100 µM $H_2O_2$. Cells were imaged every 20 minutes for 12 hours. $H_2O_2$ was added 40 minutes into the experiment. **j,m,p** Heat maps of single-cell traces of

nuclear fraction of FOXO1-mVenus (left), and nuclear p53-mCherry (right) for 12 hours following $H_2O_2$. Each row of the heat maps is a single cell over time. Both FOXO1-mVenus and p53-mCherrry heat maps are sorted by the duration that FOXO1-mVenus remained in the nucleus. Gray indicates cell death. Cells were treated with **j** 50 μM $H_2O_2$ (n=201, 6% cell death), **m** 80 μM $H_2O_2$ (n=216, 30% cell death) and **p** 100 μM $H_2O_2$ (n=254, 58% cell death. **k,n,q** Mean nuclear fraction of FOXO1-mVenus (blue, left y-axis), and p53-mcherry (red, right y-axis) for cells treated with **k** 50 μM $H_2O_2$, **n** 80 μM $H_2O_2$, and **q** 100 μM $H_2O_2$. Source data are provided in Source Data Supp Figure 2. Time-lapse imaging experiments were repeated a total of three times with similar results.

A

| Transcription Factor | Z-Score 2,3 | Z-score 6 | Family |
|---|---|---|---|
| HSF4, HSF1, HSF2 | 4.2, 3.8, 3.4 | -1.8, -1.7, -1.6 | HSF |
| FOXO3, FOXO1, FOXO6 | 3.8, 3.4, 2.0 | -1.3, -1.0, -.8 | FOX |
| GRHL1 | 2.2 | -0.9 | Grainyhead |
| REL, NFKB1, RELA, RELB | 1.8, 1.6, 1.5, 1.1 | -.2, -.4, -.4, -.3 | NF-KB |
| ZKSCAN1 | 1.5 | -1 | >3 adjacent zinc fingers |
| NFAT5, NFATC3, NFATC2 | 1.5, 1.1, .9 | -.3, -.6, -.5 | NFAT |

B

| Transcription Factor | Z-score 6 | Z-Score 2,3 | Family |
|---|---|---|---|
| JUN, JUNB, JUND, BACH1, BACH2, ATF7 | 6.8, 6.7, 6.3, 6.2, 6.0, 4.0 | -.6, -.7, -.6, -.8, -.9, -3.1 | Jun |
| JDP2, FOSL2, FOS, FOSL1 | 6.8, 6.6, 6.6, 6.4 | -.6, -.7, -.5, -.7 | FOS |
| BATF | 4.0 | -0.4 | B-ATF-related factors |
| NFE2L2 | 3.3 | -0.1 | |
| ATF4 | 3.0 | -2.3 | ATF-4-related factors |
| CREB3, CREB3L4, CREM | 2.6, 2.6, 1.7 | -2.4, -2.4, -1.7 | CREB-related factors |
| TP53 | 1.3 | 0.1 | |

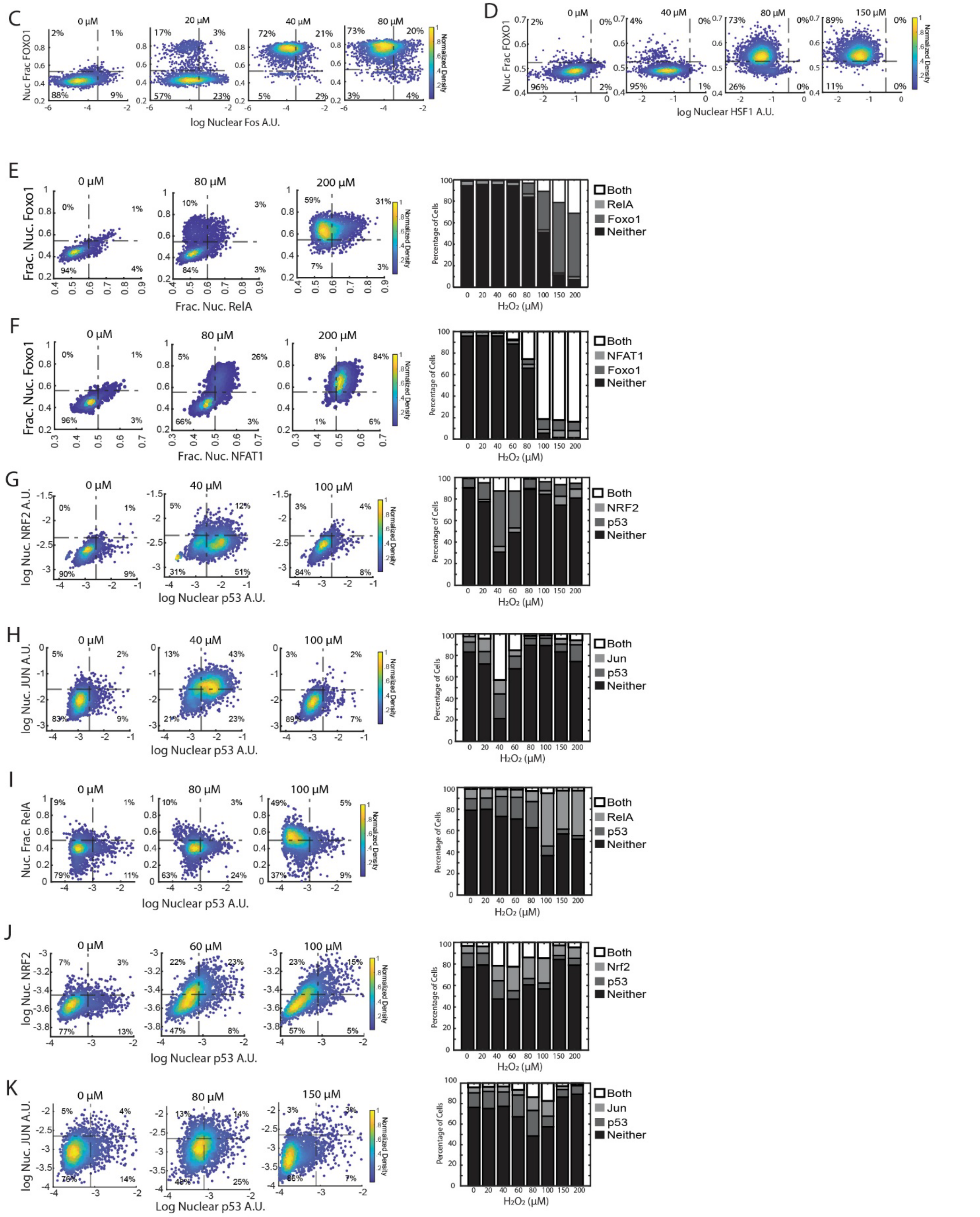

**Supplemental Fig 3: Other TFs are activated with FOXO1 and p53.** **a** TF binding motifs enriched in clusters 2 and 3 of single-cell ATAC data. **b** TF binding motifs enriched in cluster 6. Motifs of TFs not expressed in gene expression data (<.1 counts per cell) were omitted from a,b. **c-h** Immunofluorescence data measured in MCF7 cells represented as density colored scatter plots. **c** Nuclear fraction of FOXO1 and log of nuclear FOS in MCF7 cells (n>1500) treated with $H_2O_2$ for 5 hours. **d** Nuclear fraction of FOXO1 and nuclear fraction of HSF1 for MCF7 cells (n>3000) treated with $H_2O_2$ for 5 hours. **e** Fraction of RelA in the nucleus and the fraction of FOXO1 in the nucleus following $H_2O_2$ treatment for 5 hours. MCF7 cells (n>5000). **f** Fraction of NFAT1 in the nucleus and the fraction of FOXO1 in the nucleus following $H_2O_2$ treatment for 5 hours. MCF7 cells (n>5000). **g** log nuclear p53 and log nuclear NRF2 following $H_2O_2$ treatment for 5 hours. MCF7 cells (n>1000) **h** log nuclear p53 and log nuclear JUN (y-axis) following $H_2O_2$ treatment for 5 hours. MCF7 cells (n>500) **i-k** is immunofluorescence data measured in MCF10A cells. **i** log nuclear p53 and the fraction of RelA in the nucleus following $H_2O_2$ treatment for 2 hours. (n>2000) **j** log nuclear p53 and log nuclear NRF2 following $H_2O_2$ treatment for 2 hours. (n>1500) **k** log nuclear p53 and log nuclear JUN (y-axis) following $H_2O_2$ treatment for 2 hours (n>1500). Source data are provided in Source Data Supp Figure 3. Source data for all single-cell ATAC data is provided here: https://www.ncbi.nlm.nih.gov/geo/query/acc.cgi?acc=GSE227556. All immunofluorescence experiments have been repeated three times with similar results.

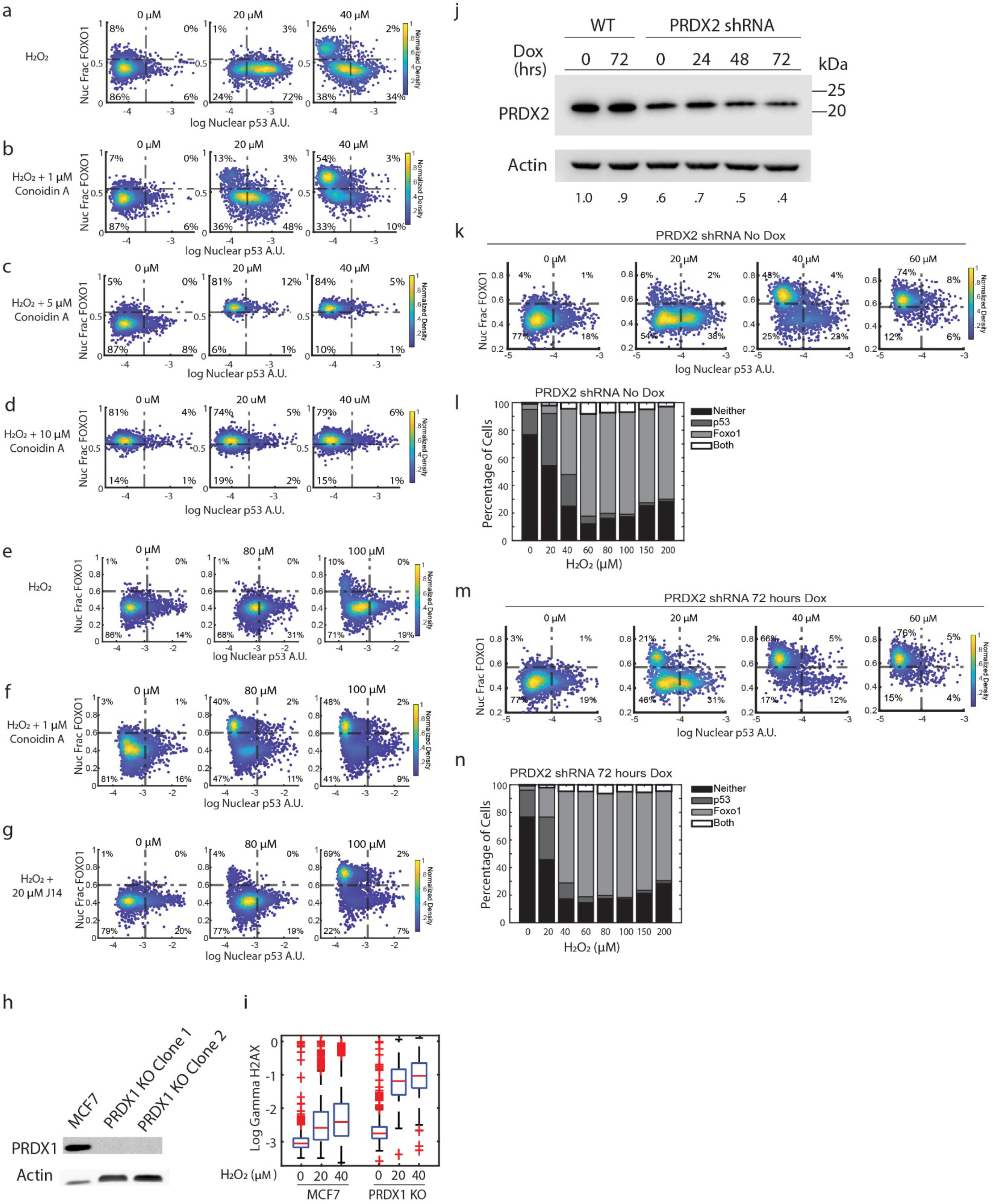
a
b
c
d
e
f
g
h
i
j
k
l
m
n
H2O2
H2O2 + 1 μM Conoidin A
H2O2 + 5 μM Conoidin A
H2O2 + 10 μM Conoidin A
H2O2 + 20 μM J14
0 μM
20 μM
40 μM
60 μM
80 μM
100 μM
Nuc Frac FOXO1
log Nuclear p53 A.U.
Normalized Density
MCF7
PRDX1 KO Clone 1
PRDX1 KO Clone 2
PRDX1
Actin
Log Gamma H2AX
H2O2 (μM)
PRDX1 KO
WT
PRDX2 shRNA
Dox (hrs)
0 72 0 24 48 72
kDa
PRDX2
25
20
1.0 .9 .6 .7 .5 .4
PRDX2 shRNA No Dox
PRDX2 shRNA 72 hours Dox
Percentage of Cells
Neither
p53
Foxo1
Both
H2O2 (μM)

**Supplemental Figure 4: The PRDX/SRXN1 system controls p53 and FOXO1 activation. a-d** Density colored scatter plots of log nuclear levels of p53 (x-axis) and the nuclear fraction of FOXO1 (y-axis) in MCF7 cells (n>1000) treated with **a** $H_2O_2$, or $H_2O_2$ combined with **b** 1 μM Conoidin A, **c** 5μM Conoidin A, **d** 10 μM of Conoidin A. Measure by immunofluorescence after 5 hours of treatment. **e-g** Density colored scatter plots of MCF10A cells (n>2000) treated with **e** $H_2O_2$, or $H_2O_2$ combined with **f** 1 μM Conoidin A, **g** 20 μM J14. Measured by immunofluorescence 2 hours after treatment. **h** Western Blot stained with PRDX1 antibody to validate PRDX1 knockout MCF7 cells. **i** Bar and whisker plots of γH2AX levels. Measured by immunofluorescence (n>2000) in MCF7 cells and MCF7 PRDX1 knockout cells treated with 20μM of $H_2O_2$ for 3 hours. Central line indicates the median. Bottom and top edges of the box indicate the 25$^{th}$ and 75$^{th}$ percentiles. Whiskers indicate the extreme data points. Outliers are indicated by a red plus sign. **j** Western blot of PRDX2 levels of MCF7 cells (WT) and MCF7 cells harboring a doxycycline (Dox) inducible shRNA targeting PRDX2. Actin is the loading control. Time of Dox exposure in hours is indicated above the blot. Quantification at bottom is PRDX2 levels divided by actin levels normalized to WT cells at 0 hours. **k** Density colored scatter plots of nuclear p53 levels and the nuclear fraction of FOXO1 of MCF7 PRDX2 shRNA cells without doxycycline. Cells (n>900) treated with $H_2O_2$ for 5 hours. Data from immunofluorescence experiments. **l** Same experiment as K; bar graph indicating the percentage of cells activating p53 and FOXO1 for all concentrations of $H_2O_2$ tested. **m** Density colored scatter plots of p53 levels and the nuclear fraction of FOXO1 of MCF7 PRDX2 shRNA cells treated with doxycycline for 72 hours before $H_2O_2$. $H_2O_2$ treatment for 5 hours. (n>1500) **n** Same experiment as m; bar graph indicating the percentage of cells activating p53 and FOXO1 for all $H_2O_2$ concentrations. Doxycycline concentrations for all experiments is 1.25 μg/ml. Source data are provided in Source Data Supp Figure 4. Immunofluorescence experiments and western blots repeated three times with similar results.

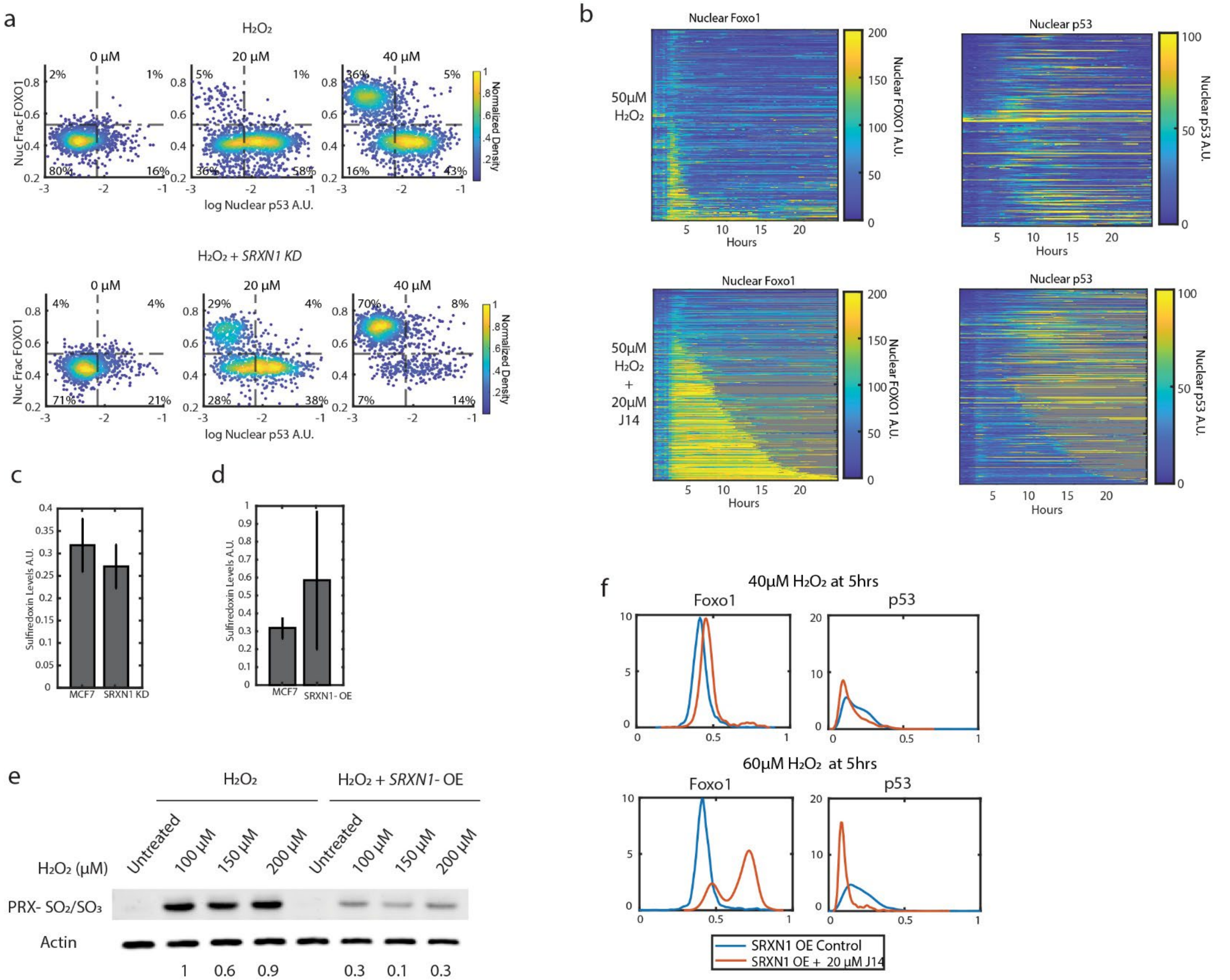


**Supplemental Figure 5: Sulfiredoxin shortens the time that FOXO1 is activated. a** Density scatter plots of MCF7 cells treated with $H_2O_2$ (top) and MCF7 cells with a SRXN1 knockdown (bottom, *SRXN1* KD) treated with indicated concentrations of $H_2O_2$ for 3 hours. (n>1000) **b** Heatmaps of single cell traces of the nuclear FOXO1-mVenus (left) and nuclear p53-mCherry (right) in MCF7 cells treated with 50 μM $H_2O_2$ (top, n=306, 13% cell death) and 50μM $H_2O_2$ + 20μM J14 (n=406, 70% cell death) for 24 hours. Each row of the heat map is a single cell over time, images were captured every 20 minutes. Both FOXO1 and p53 heat maps were sorted by the duration that FOXO1-mVenus remained in the nucleus. Gray indicates cell death. **c** Bar graphs of mean Sulfiredoxin levels and standard deviation (error bars) in cells with the shRNA knockdown compared to the MCF7 control. **d** Bar graphs of mean levels of Sulfiredoxin and standard deviation (error bars, n> 1000) in cells with *SRXN1* overexpression compared to MCF7 cells using immunofluorescence. **e** Western blot of MCF7 cells and *SRXN1* overexpressing (*SRXN1*-OE) cells treated with indicated concentrations of $H_2O_2$ for 3 hours and stained for hyperoxidized ($SO_2/SO_3$) PRDX1/2 and Actin. **f** Population density plots of nuclear fraction of FOXO1 and nuclear p53 levels in cells overexpressing Sulfiredoxin (*SRXN1*-OE) after treatment with $H_2O_2$ and the same cells treated with 20μM of J14 at indicated concentrations of $H_2O_2$. Blue line indicates cells with overexpression of *SRXN1* and the red line indicates cells overexpressing *SRXN1* treated with 20μM J14. Source data are provided in Source Data Supp Figure 5. All immunofluorescence and western blot experiments were repeated three times with similar results.

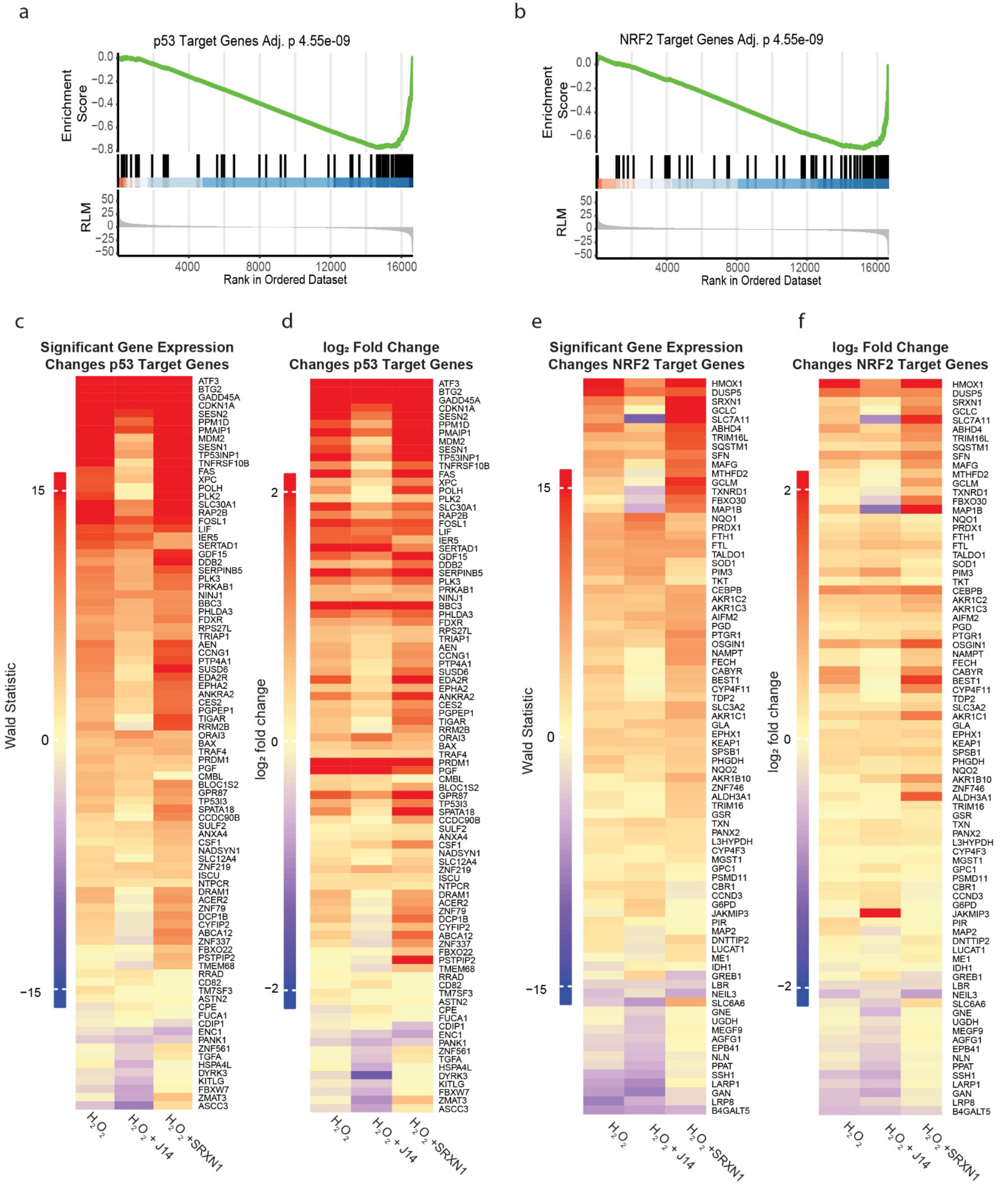


**Supplemental Figure 6. Differential gene expression analysis of p53 and NRF2 target genes using RNA-seq. a** Gene set enrichment analysis (GSEA) of p53 target genes in J14 + $H_2O_2$ samples as compared to SRXN1-

OE + $H_2O_2$ samples **b** GSEA of NRF2 target genes in J14 + $H_2O_2$ samples as compared to SRXN1-OE + $H_2O_2$ samples. **c** Wald statistic and **d** $log_2$ fold changes of p53 target genes in $H_2O_2$ treated cells vs. PBS controls, $H_2O_2$ + J14 treated cells vs J14 treated controls and $H_2O_2$ treated SRXN1-OE cells vs. SRXN1-OE PBS controls. **e** Wald statistic and **f** $log_2$ fold changes of NRF2 target genes in $H_2O_2$ treated cells vs. PBS controls, $H_2O_2$ + J14 treated cells vs J14 treated controls and $H_2O_2$ treated SRXN1-OE cells vs. SRXN1-OE PBS controls. $H_2O_2$ concentration is 50µM, J14 20µM. (RLM, Ranked List Metric). P-values in a and b were calculated by permutation test and adjusted using the Benjamini-Hochberg correction. RNA-seq was performed on 3 biological replicates. Source data are available here: https://www.ncbi.nlm.nih.gov/geo/query/acc.cgi?acc=GSE227556.
