# Supplementary material for: Temporal coordination of the transcription factor response to H2O2 stress
Source: Nat Commun. 2024 Apr 23;15:3440. doi: 10.1038/s41467-024-47837-w (PMC11039679; doi:10.1038/s41467-024-47837-w)
Supplement: Supplementary file 3 — Description of Additional Supplementary Files [file 41467_2024_47837_MOESM3_ESM.pdf]

File name: Supplementary Movie 1
Description: Example movie showing FOXO1-mVenus and p53-mCherry activity after treatment with 50μM of H_2O_2 in MCF7 cells for a period of 24hrs.H_2O_2 was added at the 20-minute timepoint.

File name: Supplementary Movie 2
Description: Example movie showing FOXO1-mVenus and p53-mCherry activity after treatment with 80μM of H_2O_2 in MCF7 cells for a period of 24hrs.H_2O_2 was added at the 20-minute timepoint.

File name: Supplementary Movie 3
Description: Example movie showing FOXO1-mVenus and p53-mCherry activity after treatment with 100μM of H_2O_2 in MCF7 cells for a period of 24hrs.H_2O_2 was added at the 20-minute timepoint.

File name: Supplementary Movie 4
Description: Example movie showing FOXO1-mVenus and p53-mCherry activity after treatment with 300μM of H_2O_2 in MCF7 cells for a period of 24hrs.H_2O_2 was added at the 20-minute timepoint.
