# Supplementary material for: Temporal coordination of the transcription factor response to H2O2 stress
Source: Nat Commun. 2024 Apr 23;15:3440. doi: 10.1038/s41467-024-47837-w (PMC11039679; doi:10.1038/s41467-024-47837-w)
Supplement: Supplementary file 8 — Reporting Summary [file 41467_2024_47837_MOESM8_ESM.pdf]

nature portfolio

Corresponding author(s): *Double-anonymous peer review submissions: write DAPR and your manuscript number here instead of author names.*

Last updated by author(s): *YYYY-MM-DD*

Reporting Summary

Nature Portfolio wishes to improve the reproducibility of the work that we publish. This form provides structure for consistency and transparency in reporting. For further information on Nature Portfolio policies, see our Editorial Policies and the Editorial Policy Checklist.

Statistics

For all statistical analyses, confirm that the following items are present in the figure legend, table legend, main text, or Methods section.

n/a	Confirmed	
☐	☒	The exact sample size (*n*) for each experimental group/condition, given as a discrete number and unit of measurement
☐	☒	A statement on whether measurements were taken from distinct samples or whether the same sample was measured repeatedly
☒	☐	The statistical test(s) used AND whether they are one- or two-sided<br>*Only common tests should be described solely by name; describe more complex techniques in the Methods section.*
☒	☐	A description of all covariates tested
☒	☐	A description of any assumptions or corrections, such as tests of normality and adjustment for multiple comparisons
☐	☒	A full description of the statistical parameters including central tendency (e.g. means) or other basic estimates (e.g. regression coefficient) AND variation (e.g. standard deviation) or associated estimates of uncertainty (e.g. confidence intervals)
☐	☒	For null hypothesis testing, the test statistic (e.g. *F*, *t*, *r*) with confidence intervals, effect sizes, degrees of freedom and *P* value noted<br>*Give P values as exact values whenever suitable.*
☒	☐	For Bayesian analysis, information on the choice of priors and Markov chain Monte Carlo settings
☒	☐	For hierarchical and complex designs, identification of the appropriate level for tests and full reporting of outcomes
☒	☐	Estimates of effect sizes (e.g. Cohen's *d*, Pearson's *r*), indicating how they were calculated

Our web collection on statistics for biologists contains articles on many of the points above.

Software and code

Policy information about availability of computer code

Data collection	For collection of data for immunofluorescence (IF) and time lapse movies NIS Elements AR 5.21.02 software and NIS Elements Viewer 5.21 from NIkon.
Data analysis	Data analysis for IF was done using Cell Profiler Version 3.1.9. Time lapse movie data was analyzed using p53CinemaManual a tracking software already published (Reyes, José et al. "Fluctuations in p53 Signaling Allow Escape from Cell-Cycle Arrest." Molecular cell vol. 71,4 (2018): 581-591.e5. doi:10.1016/j.molcel.2018.06.031). p53CinemaManual GitHub- https://github.com/balvahal/p53CinemaManual. Further analysis of the data was done using MATLAB Version R2021a. Custom MATLAB scripts were written and available on request.<br>Data for Single Cell ATAC sequencing was analyzed using the ArchR package (v. 1.0.1). BAM files were prepared by NovoGene and processed with the Rsubread package (v. 2.12.3). Data for bulk RNA sequencing was analyzed using the DESeq2 package (v. 1.38.3), GSEA was performed using the ClusterProfiler R package (v. 4.6.2), and enrichplot(v. 1.18.4).

For manuscripts utilizing custom algorithms or software that are central to the research but not yet described in published literature, software must be made available to editors and reviewers. We strongly encourage code deposition in a community repository (e.g. GitHub). See the Nature Portfolio guidelines for submitting code & software for further information.

Data

Policy information about availability of data

All manuscripts must include a data availability statement. This statement should provide the following information, where applicable:

- Accession codes, unique identifiers, or web links for publicly available datasets
- A description of any restrictions on data availability
- For clinical datasets or third party data, please ensure that the statement adheres to our policy

Data from single-cell Assay for Transposase-Accessible Chromatin using sequencing (ATAC-seq) and gene expression using the 10X genomics single-cell Multiome kit, as well as bulk RNA-seq data is available on NCBI's Gene Expression Omnibus (GEO) database accession number: GSE227556. Other source data for graphs in the Figures and Supplementary Figures is provided in the Source Data Files.

Research involving human participants, their data, or biological material

Policy information about studies with human participants or human data. See also policy information about sex, gender (identity/presentation), and sexual orientation and race, ethnicity and racism.

Reporting on sex and gender	N/A
Reporting on race, ethnicity, or other socially relevant groupings	N/A
Population characteristics	N/A
Recruitment	N/A
Ethics oversight	N/A

Note that full information on the approval of the study protocol must also be provided in the manuscript.

Field-specific reporting

Please select the one below that is the best fit for your research. If you are not sure, read the appropriate sections before making your selection.

☒ Life sciences ☐ Behavioural & social sciences ☐ Ecological, evolutionary & environmental sciences

For a reference copy of the document with all sections, see nature.com/documents/nr-reporting-summary-flat.pdf

Life sciences study design

All studies must disclose on these points even when the disclosure is negative.

Sample size	For immunofluorescence experiments we analyzed >= 2000 cells per condition. This number of cells is easily obtained in 96 well plates and is more than sufficient to capture population distributions of protein abundance by immunofluorescence. For time-lapse imaging experiments we analyzed 150-300 cells per condition. This number of cells is sufficient to capture the timing of transcription factor activation. For single cell ATAC sequencing we analyzed >= 10,0000 cells. The number of cells sequenced was chosen to obtain broad coverage while minimizing single cell doublets in the 10X Multiome protocol.
Data exclusions	For time-lapse microscopy heat maps in Figure 2 and 3, cells with high p53 levels before H2O2 treatment were excluded from the heat maps for clarity but included in all other analysis. These were less than 5% of cells.
Replication	All immunofluorescence experiments were replicated at least 3 times with reproducible results . Time lapse movies were run at least 3 times and the movies with the right cell number that clearly showed the two phases described were selected. As long as cell number was maintained the data are reproducible as the impact of H2O2 exposure decreases with cell number. Single cell ATAC sequencing was carried out once as the cost of the experiments are limiting. Moreover key findings of the single-cell ATAC data were validated using immunofluorescence. Bulk RNA sequencing was performed in triplicate for each condition. Western blots were replicated at least twice for each experiment.
Randomization	Randomization was not carried out as it was not relevant to the study. The study was performed on cell lines so randomization of treatment vs control group did not make sense.
Blinding	Blinding was not carried out for this study as it would be impractical and not improve the validity of the study

Reporting for specific materials, systems and methods

We require information from authors about some types of materials, experimental systems and methods used in many studies. Here, indicate whether each material, system or method listed is relevant to your study. If you are not sure if a list item applies to your research, read the appropriate section before selecting a response.

Materials & experimental systems

n/a	Involved in the study
☐	☒ Antibodies
☐	☒ Eukaryotic cell lines
☒	☐ Palaeontology and archaeology
☒	☐ Animals and other organisms
☒	☐ Clinical data
☒	☐ Dual use research of concern
☒	☐ Plants

Methods

n/a	Involved in the study
☒	☐ ChIP-seq
☒	☐ Flow cytometry
☒	☐ MRI-based neuroimaging

Antibodies

Antibodies used

Primary antibodies for IF: Anti-FOXO1 (C29H4) from Cell Signaling Cat# 2880S (1:500), Anti-p53 (DO-1) from Santa Cruz Cat# sc-126 (1:500), Anti-Sulfiredoxin from Santa Cruz Cat# sc-166786 (1:100), Recombinant Anti-Peroxiredoxin 1/PAG antibody [EPR5433] (ab109498) (1:1000) , NRF2 (D1Z9C) XP Rabbit mAb Cat#12721 (1:500), NFAT1 (D43B1) XP Rabbit mAb #5861 (1:300), NF-kB p65 (D14E12) XP Rabbit mAb #8242 (1:400), Anti-HSF1 antibody 10H8 Cat# SMC-118D from Stress Marq Biosciences (1:200), c-Fos (9F6) Rabbit mAb # 2250 (1:1000), and c-Jun (60A8) Rabbit mAb #9165 (1:400) from Cell Signaling.
Primary antibodies for Western Blotting: Anti-Peroxiredoxin-SO3 antibody (ab16830) (1:1000), Recombinant Anti-Peroxiredoxin 1/PAG antibody [EPR5433] (ab109498) (1:1000) from Abcam, and Actin Cat#A2228 clone AC-74 (Sigma)
Secondary antibodies used for IF: Goat Anti-Rabbit IgG (H+L) Alexa Fluor 488 Cat# A-11034, Goat Anti- Mouse IgG (H+L) Alexa Fluor 594 Cat#A-11032, Goat Anti-Rabbit IgG (H+L) ALexa Fluor 546 Cat# A-11010, Goat Anti- Mouse IgG (H+L) Alexa Fluor 647 Cat# A21236 all obtained from Invitrogen.
Secondary antibodies used for Western Blot: Goat anti mouse 680LT secondary (LICOR-IRDye) Cat# 925-68020 and Goat anti Rabbit 800CW secondary (LICOR-IRDye) Cat# 925-3221, both at 1:10000 concentration both obtained from LI-COR Biosciences.

Validation

We have validated the following antibodies with knockout/knockdown experiments in MCF7 cells: Anti-FOXO1 (C29H4) Cell Signaling Cat# 2880S, Anti-p53 (DO-1) Santa Cruz Cat# sc-126, Anti-Peroxiredoxin 1/PAG antibody [EPR5433] (ab109498) and Recombinant Anti-Peroxiredoxin 2/PRP antibody [EPR5154] (ab109367).
The Sulfiredoxin antibody from Santa Cruz Cat# sc-166786 was validated by the company by overexpressing SRXN1 in 293T cells. The NRF2 (D1Z9C) XP Rabbit mAb Cell Signaling Cat#12721 antibody was validation by the company with NRF2 knock-out MEF cells. Anti-HSF1 antibody 10H8 Cat# SMC-118D from Stress Marq Biosciences was validated by the company using knock-out MEF cells and in mouse lung tissue. The c-Jun (60A8) Rabbit mAb #9165 (1:400) from Cell Signaling was validated using knock-out HeLa cells.

Eukaryotic cell lines

Policy information about cell lines and Sex and Gender in Research

Cell line source(s)

MCF7 cell line was a gift from Galit Lahav, Harvard Medical School. Other cell lines A549 (CCL-185), U-2 OS (HTB-96) and MCF10A (CRL-10317) were obtained from ATCC.

Authentication

Cell line authentication: A549, U-2 OS and MCF10A cells were validated by ATCC by short tandem repeat profiling. MCF7 cells were validated by short tandem repeat profiling by the University of Arizona Genetics Core.

Mycoplasma contamination

All cell lines were tested for mycoplasma and no contamination was found.

Commonly misidentified lines
(See ICLAC register)

No misidentified cell lines were used.

Plants

Seed stocks	N/A
Novel plant genotypes	N/A
Authentication	N/A
